# The effect of incident tuberculosis on immunological response of HIV patients on highly active anti-retroviral therapy at the university of Gondar hospital, northwest Ethiopia: a retrospective follow-up study

**DOI:** 10.1186/1471-2334-14-468

**Published:** 2014-08-27

**Authors:** Abate Assefa, Baye Gelaw, Gebeyaw Getnet, Gashaw Yitayew

**Affiliations:** Department of Medical Microbiology, College of Medicine and Health Sciences, School of Biomedical and Laboratory Sciences, University of Gondar, Gondar, Ethiopia; Department of Medical Parasitology, College of Medicine and Health Sciences, School of Biomedical and Laboratory Sciences, University of Gondar, Gondar, Ethiopia; Bahir Dar Regional Laboratory institute, Bahir Dar, Ethiopia

**Keywords:** Anti-retroviral therapy, Immunological failure, Incident TB

## Abstract

**Background:**

Human immunodeficiency virus (HIV) infection is usually complicated by high rates of tuberculosis (TB) co-infection. Impaired immune response has been reported during HIV/TB co-infection and may have significant effect on anti-retroviral therapy (ART). TB/HIV co - infection is a major public health problem in Ethiopia. Therefore, the aim of the study was to assess the effect of TB incidence on immunological response of HIV patients during ART.

**Methods:**

A retrospective follow-up study was conducted among adult HIV patients who started ART at the University of Gondar Hospital. Changes in CD4+ T - lymphocyte count and incident TB episodes occurring during 42 months of follow up on ART were assessed. Life table was used to estimate the cumulative immunologic failure. Kaplan-Meier curve was used to compare survival curves between the different categories. Cox-proportional hazard model was employed to examine predictors of immunological failure.

**Results:**

Among 400 HIV patients, 89(22.2%) were found to have immunological failure with a rate of 8.5 per 100 person-years (PY) of follow-up. Incident TB developed in 26(6.5%) of patients, with an incidence rate of 2.2 cases per 100 PY. The immunological failure rate was high (20.1/100PY) at the first year of treatment. At multivariate analysis, Cox regression analysis showed that baseline CD4+ T - cell count <100 cells/mm^3^ (adjusted hazard ratio (AHR) 1.8; 95%CI: 1.10 - 2.92, p = 0.023) and being male sex (AHR 1.6; 95%CI: 1.01 - 2.68, p = 0.046) were found to be significant predictors of immunological failure. There was borderline significant association with incident TB (AHR 2.2; 95%CI: 0.94 - 5.09, p = 0.06). The risk of immunological failure was significantly higher (38.5%) among those with incident TB compared with TB - free (21.1%) (Log rank p = 0.036).

**Conclusions:**

High incidence of immunological failure occurred within the first year of initiating ART. The proportions of patients with impaired immune restoration were higher among patients with incident TB. Lower baseline CD4+ T - cells count of <100 cells/mm^3^ and being male sex were significant predictors of immunological failure. The result highlighted the beneficial effects of earlier initiation of ART on CD4+ T - cell count recovery.

**Electronic supplementary material:**

The online version of this article (doi:10.1186/1471-2334-14-468) contains supplementary material, which is available to authorized users.

## Background

Despite recent advances in anti**-**retroviral therapy (ART), human immunodeficiency virus (HIV) infections and the resulting acquired immunodeficiency syndrome (AIDS) remain an important cause of morbidity and mortality worldwide with 2.6 million new cases and 1.8 million deaths by the year 2009 [1]. In Ethiopia, according to the 2007 single point HIV prevalence estimate, there were 1,216,908 adult people living with HIV (PLHIV), and of these 397,818 expected to take ART treatment by the year 2010 [2]. On the other hand, tuberculosis (TB) caused by *Mycobacterium tuberculosis*, remains the leading causes of death from infectious diseases worldwide. In 2012, about 8.6 million incident TB and 1.3 million deaths due to TB were reported globally. The majority of TB cases occurred in Asian (58%) and African (27%) countries [3].

In developing countries, TB remains a major public health threat among HIV-infected individuals [3, 4]. HIV is the most potent risk factor for TB and TB is the leading cause of morbidity and mortality in HIV/AIDS patients [5, 6]. Tuberculosis enhances progression of HIV infection and HIV increases the risk of infection as well as reactivation of latent tuberculosis. It is estimated that 50 - 60% of PLHIV will develop TB disease in their lifetime in contrast with HIV negative persons, whose lifetime risk is only 10% [4, 7]. The proportion of TB cases co-infected with HIV is highest in African countries. In African countries, about 37% of TB cases were co-infected with HIV which accounted for 75% of TB cases among HIV positive people worldwide [3]. In 2007, based on Federal HIV/AIDS Prevention and Control Office report in Ethiopia, the TB/HIV co**-**infection rate was 20 **-** 50% [8]. According to WHO report, in 2012 the incidence of TB infection in Ethiopia was 247 per 100,000 people and 10.2% of them were estimated to have co-infection with HIV [3].

With the advent of ARV drugs, HIV/AIDS has become a treatable chronic disease. Effective anti-retroviral therapy (ARV) therapy is usually convoyed by an increase in the number of CD4+ T **-** cells and the functional restoration of patents’ immune response and decline in HIV viral load as well. However, the requirement of regular and lifelong medication of HIV patient is challenged with emergencies of treatment failure [8–10]. Impaired immunological recovery may indicate incomplete suppression of plasma HIV **-** RNA which results ARV drug resistance [9]. HIV treatment failure can be defined as progression of disease after ART initiation. Anti**-**retroviral treatment failure can be assessed clinically, immunologically and virologically. However, on the basis of clinical criteria treatment failure can’t be concluded. Despite viral load testing is preferred approach for monitoring ART response, in resource limited settings, immunological failure criteria, trends in CD4+ T **-** cells counts over time, remains the strongest predictor of treatment failure [8].

The recovery of CD4+ T **-** cells count during ART in HIV/TB co-infected patients is less clear. However, studies assessing immune responses to ART have found poor CD4+ T **-** cells recovery to occur in patients who develop incident TB after initiating ART [11–16]. Moreover, in spite of HIV statuses sever CD4+ T **-**lymphocytopenia has been observed in TB patients [17]. An in vitro study indicated that TB infection impairs cellular immune responses through *M. tuberculosis*-induced apoptosis of T **-** cells [18]. Therefore, TB may act as a cofactor that accelerates the impairment of the immune function and shortens survival of HIV **-** infected individuals. Although TB/HIV co-infection is a major public health problem in Ethiopia, no studies have reported the effect of TB on immunological responses of HIV patients during ART. Hence, assessing the effect of TB on immunological responses of HIV patients will provide information for clinicians for appropriate management of TB/HIV co-infected patients. Moreover, policy makers and health professionals can use the findings to design ART related programs. Therefore, the aim of this study was to assess the effect of incident TB on immunological response of HIV/AIDS patients during ART at the University of Gondar Hospital.

## Methods

### Study setting and population

The study was conducted at the University of Gondar Hospital, North-west Ethiopia, in June 2013. University of Gondar Hospital is a referral hospital with more than 400 beds serving a population of about 5 million people in North-west Ethiopia. The hospital offers a wide range of services including voluntary counseling and testing, treatment, referral services, monitoring of treatment response with CD4+ T **-** cells counts, follow-up and supportive care of HIV-infected individuals. ART is provided free of cost to all HIV **-** infected individuals in need of treatment. The study populations were those patients who are 15 years old and above and ever started ART at the University of Gondar Hospital. Patients start ART based on WHO criteria for the initiation of ART in adults and adolescents; patients with CD4+ T **-** cell count <200 cell/mm^3^ or WHO stage 4 irrespective of CD4+ T **-** cell count or WHO stage 3 with CD4+ T **-** cell counts <350 cells/mm^3^. Routine monitoring of CD4+ T **-** cell counts is performed every six months, or more frequently if clinically indicated [8]. All HIV patients were screened for TB at enrolment, and during each follow**-**up visit through clinical evaluation and microbiological tests and chest-radiography based on WHO national guidelines. Patients diagnosed with incident TB were treated with a standard eight months regimen with two months intensive phase, combination of 4 drugs (isoniazid, rifampicin, ethambutol and pyrazinamide), and a subsequent 6 months continuation phase, 2 drugs (isoniazid, ethambutol) [4]. Routine CD4+ T **-** lymphocyte count measurements were performed by FACS count (Becton Dickinson) every 6 months or more frequently if clinically indicated after initiating ART.

### Study design and data collection

A retrospective follow-up study was conducted on patients initiating ART from the 1st of September 2007 and 30th of August 2008 at the University of Gondar Hospital. This period was selected to collect patient’s information from sufficient follow**-**up time. Adult HIV patients whose charts were available and who had at least 6 months of follow **-** up (having at least two CD4+ T**-**cell measurements) and started on first line ART during the study period were eligible for the study. HIV/AIDS patients who had active TB (prevalent TB) at the initiation of ART and missing charts or incomplete baseline and follow-up data were excluded from the study. Changes in CD4+ T **-** cells count and incident TB episodes occurring during the 42 months of follow-up on ART were assessed. Socio **-** demographic information of the patients and clinical characteristics such as time of ART start, baseline and follow-up CD4+ T **-** cell count, and WHO clinical stage, co**-**infection with TB, time of TB development and functional status were extracted from the hospital’s ART register. Baseline measurements of CD4+ T **-** cell count was included if performed within six months prior to ART initiation and once on treatment follow**-**up measurements was included if performed within ±2 months of the 6 month time points. Data extraction format was prepared and pretested on 20 charts. The data were extracted from patients’ charts by three nurses who had ART training and experience in HIV care.

### Definitions

Prevalent TB was defined as patients taking anti-TB treatment at the time of starting ART. Incident TB was defined as a new active TB developed after initiation of ART. Immunological failure was defined based on WHO criteria: decrease in CD4+ T **-** cell count to pre **-**ART level or below, decrease in CD4+ T **-** cell count from on-treatment peak value by more than 50% or persistent CD4+ T **-** cell count <100 cells/mm^3^ after six months of therapy [8]. Patients, who were died, transfer out or, lost **-** to **-** follow**-**up or didn’t show the event until the last visit was considered as censored.

### Statistical methods

Immunologic failure rates were calculated per 100 person years at risk. Data were entered and cleaned by using Epi**-**Info version.3.5.3 then exported to Statistical Package for the Social Sciences (SPSS) version 20. Descriptive analyses were used to determine baseline socio-demographic and clinical characteristics of the patients. Life table was used to estimate the cumulative probabilities of immunologic failure. Kaplan**-**Meier survival curve was used to estimate the median survival time from initiation of ART to immunologic failure. Bivariate and multivariate Cox-proportional hazard model was employed to identify predictors of immunological failure. Hazard Ratio (HR) with 95% confidence intervals were computed and p-value < 0.05 was considered statistically significant for all cases.

### Ethical approval

The study was reviewed and approved by the Institutional Review Board (IRB) of the University of Gondar. Official permission was obtained from University of Gondar Hospital management. The patient records were anonymized and de**-**identified prior to analysis. Individual records were coded and accessed only by research staff.

## Results

### Baseline socio-demographic characteristics of patients

A total of 606 adult HIV/AIDS patients were newly enrolled to HIV care clinic from the 1st of September 2007 and 30th of August 2008 but 122 patients were excluded due to missing charts or incomplete baseline and follow**-**up data. Among 484 HIV patients who had at least 6 months of follow-up, 84(17.4%) had active TB at the initiation of ART and the analysis was restricted to those who did not have active TB at ART start. Therefore, a total of 400 adult HIV/AIDS patients record were analyzed. Of the 400 adult HIV/AIDS patients who met the inclusion criteria for the study, 315 (78.8%) remained within the programme at study censorship, 4 (1%) died, 67 (16.8%) transferred out and 14(3.5%) were lost to follow **-** up from the programme. Of 400 adult HIV/AIDS patients, the majority (64.5%) were females. The median age of patients at ART start was 33 years (Inter Quartile Range (IQR) = 27**-**40 years) and almost half of the patients were married (50.5%). One hundred seventeen (29.3%) of the study participants had no formal education and only 62 (15%) patients were employed (Table 1).

**Table 1:** **Baseline socio-demographic characteristics of HIV/AIDS patients who started ART from September 2007 to August 2008 at the University of Gondar Hospital**

Variable	n	%
**Gender**		
Male	142	35.5
Female	258	64.5
**Age**		
16-30	165	41.3
31-50	211	52.8
> 50	24	6.0
**Religion**		
Orthodox	364	91.0
Muslim	33	8.3
Others	3	0.8
**Residence**		
Urban	318	79.5
Rural	82	20.5
**Marital status**		
Single	63	15.8
Married	202	50.5
Divorced	87	21.8
Widowed	48	12.0
**Educational status**		
Illiterate	117	29.3
Primary	111	27.8
Secondary	129	32.3
Tertiary	43	10.8
**Occupational status**		
Employed	62	15.5
Unemployed	193	48.3
Housewife	80	20.0
Merchant	48	12.0
Farmer	17	4.3

### Baseline clinical characteristics of patients

During ART enrolment more than half (52%) of patients were at WHO clinical stage III. At the time of ART initiation, patients had advanced immunodeficiency with the median baseline CD4+ T **-** cell counts of 152 cells/mm^3^ (IQR = 82 **-** 203 cells/mm^3^). One hundred and twenty seven (31.8%) of patients had baseline CD4+ T **-** cell counts <100 cells/mm^3^. Majority of the patients started treatment with zidovudine (AZT)/lamivudine (3TC)/nevirapine (NVP) or efavirenz (EFV) (47.3%) followed by tenofobir (TDF)/3TC/NVP or EFV (40.5%) and stavudine (D4T)/3TC/NVP or EFV (12.3%) based combination first line ARV drug regimens. Three hundred and forty (85%) patients were working by their functional status (Table 2).

**Table 2:** **Baseline clinical characteristics and TB incidence of HIV/AIDS patients who started ART from September 2007 to August 2008 at the University of Gondar Hospital**

Variable	Total	Incident TB+	Incident TB-
**Female sex (n[%])**	258(64.5)	18(69.2)	240(64.2)
**Age (years, mean [SD])**	34.37 ± 9.29	35.4 ± 8.8	34.3 ± 9.3
**WHO HIV clinical stage (n[%])**			
I	57(14.3)	2(3.5)	55(96.5)
II	86(21.5)	6(7.0)	80(93.0)
III	208(52.0)	13(6.2)	195(93.8)
IV	49(12.3)	5(10.2)	44(89.8)
**CD4 count (cells/mm** ^**3**^ **, median [IQR])**	152(82, 203)	98(58,201.75)	153(86,203)
**CD4 count (cell/mm** ^**3**^ **, n[%])**			
0-99	127(31.8)	13(10.4)	112(89.6)
≥100	170(42.5)	13(4.7)	262(95.3)
**Initial ART Regimen (n[%])**			
d4T/3TC/NVP or EFV	49(12.3)	4(8.2)	45(91.8)
AZT/3TC/NVP or EFV	189(47.3)	15(7.9)	174(92.1)
TDF/3TC/NVP or EFV	162(40.5)	7(4.3)	155(95.7)
**Functional status (n[%])**			
Working	340(85.0)	20(5.9)	320(94.1)
Ambulatory	49(12.3)	4(8.2)	45(91.8)
Bed ridden	11(2.8)	2(18.2)	9(81.8)

d4T: stavudine; 3TC: lamivudine; NVP: nevirapine; AZT: zidovudine; EFV: efavirenz; TDF: tenofobir; TB: tuberculosis; HIV: human immunodeficiency virus; ART: antiretroviral therapy; IQR: interquartile range; SD: standard deviation; WHO: world health organization.

### Immunological failure after initiation of ART

**Figure 1:**
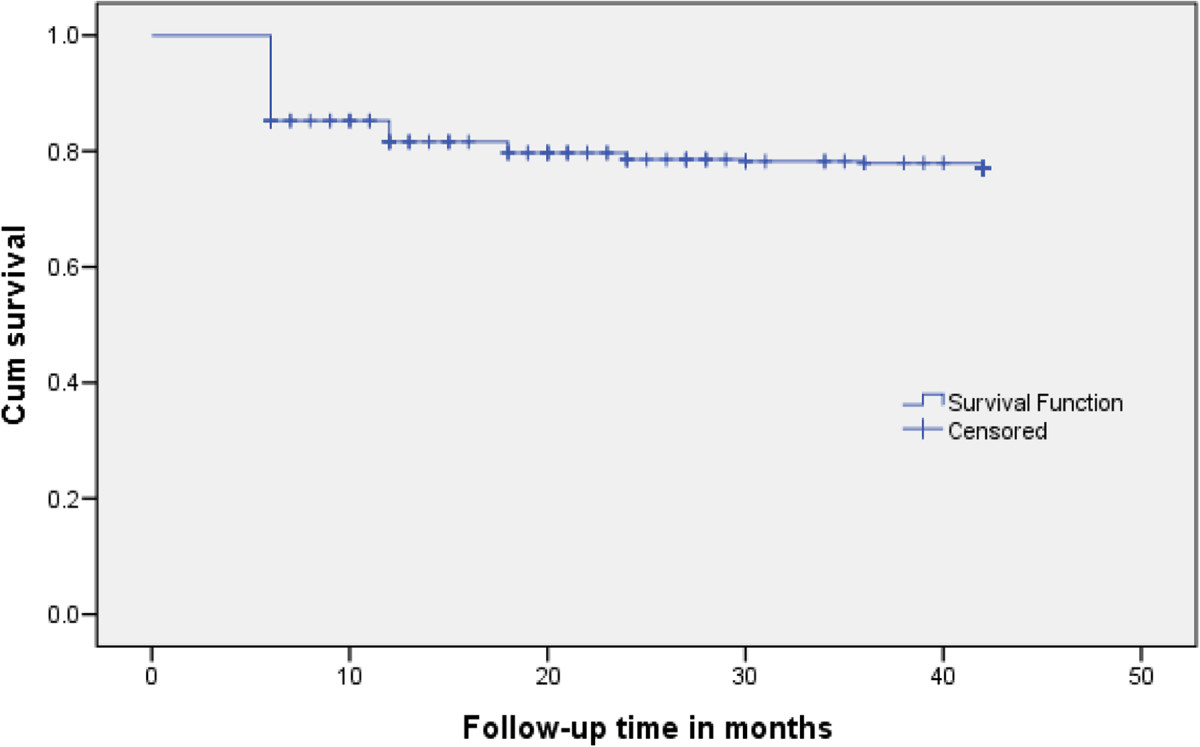
**Kaplan-Meier curve for immunological failure of HIV/AIDS patients taking ART.** The Kaplan-Meier curve indicated the trends of cumulative survival of patients from immunological failure within 42 months of follow up on ART.

Study participants were followed for a minimum of 6 and a maximum of 42 months with total person-time follow-up of 1042.7 person-years (PY). The median number of CD4+ T **-** cell counts performed per patient during the follow **-**up period was 7 (IQR = 6 **-** 8). Of the 400 HIV/AIDS patients who had at least 6 months of follow **-**up, 85(21.3%) patients were lost to follow **-** up before the study censorship. Eighty nine (22.2%) of the patients were found to have immunological failure. Based on WHO criteria, 49(55%) immunological failure were defined by decrease in CD4+ T **-** cell count to pre **-**ART level or below, 12(13.5%) decrease in CD4+ T **-** cell count from on**-**treatment peak value by more than 50% and 28 (31.5%) persistent CD4+ T **-** cell count below 100 cells/mm^3^. The median time for occurrence of immunological failure was 6 months (IQR = 6 **-** 12 months). The median time for immunological failure was 6 months for both TB and TB**-**free cohorts. Among all 89 immunological failures, 60(67.4%) patients were failed at 6 month of follow-up on ART. The overall immunological failure rate of our cohort was 30 per 100PY at the end of 6 months, 20.1 per 100 PY at the end of one year, 16.2 per 100 PY at the end of 18 months, 13.0 per 100 PY at the end of two years, 11.1 per 100 PY at the end of 30 months, 10.8 per 100 PY at the end of three years and 8.5 per 100 PY at the end of 42 months of follow-up. The cumulative probability of survival of patients from immunological failure at the end of 6 months was 85% while at the end of one year was 79%, at the end of three years 78% and at the end of 42 months 75% (Figure 1).

**Figure 2:**
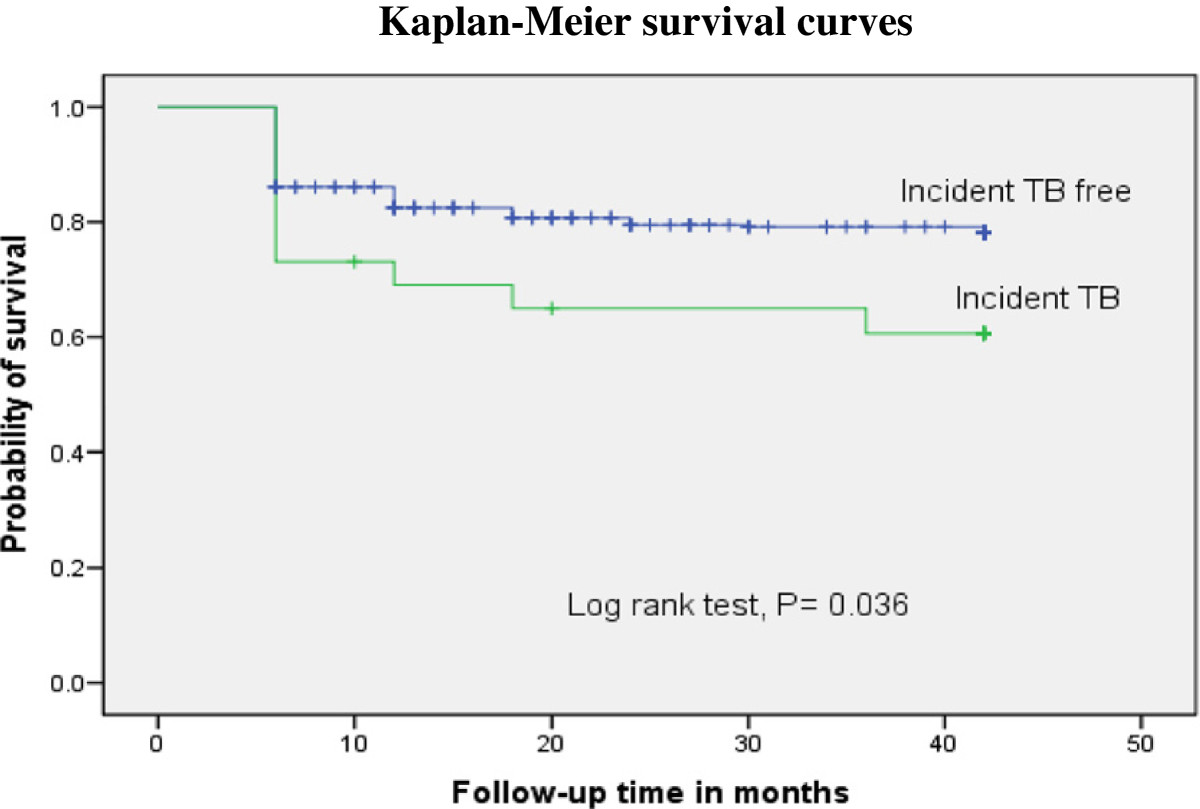
**Kaplan-Meier curve illustrating the probability of immunological failure survival to development of incident TB.** The immunological failure survival was lower in patients with active TB as compared to those patients remaining TB - free.

Active TB developed in 26(6.5%) patients within 42 months on ART, with an incidence rate of 2.2 cases per 100 PY of follow**-**up. The median time for development of incident TB during the follow-up was 9.5 months (IQR, 5.5 **-** 16.5 months). Among those who developed TB during the follow**-**up time, 17(57.7%) of the incident TB occurred within the first year of initiating ART. After 42 months on ART, the risk of immunologic failure was 38.5% versus 21.1% among patients with and without incident TB respectively (log rank p = 0.036) (Figure 2).

**Figure 3:**
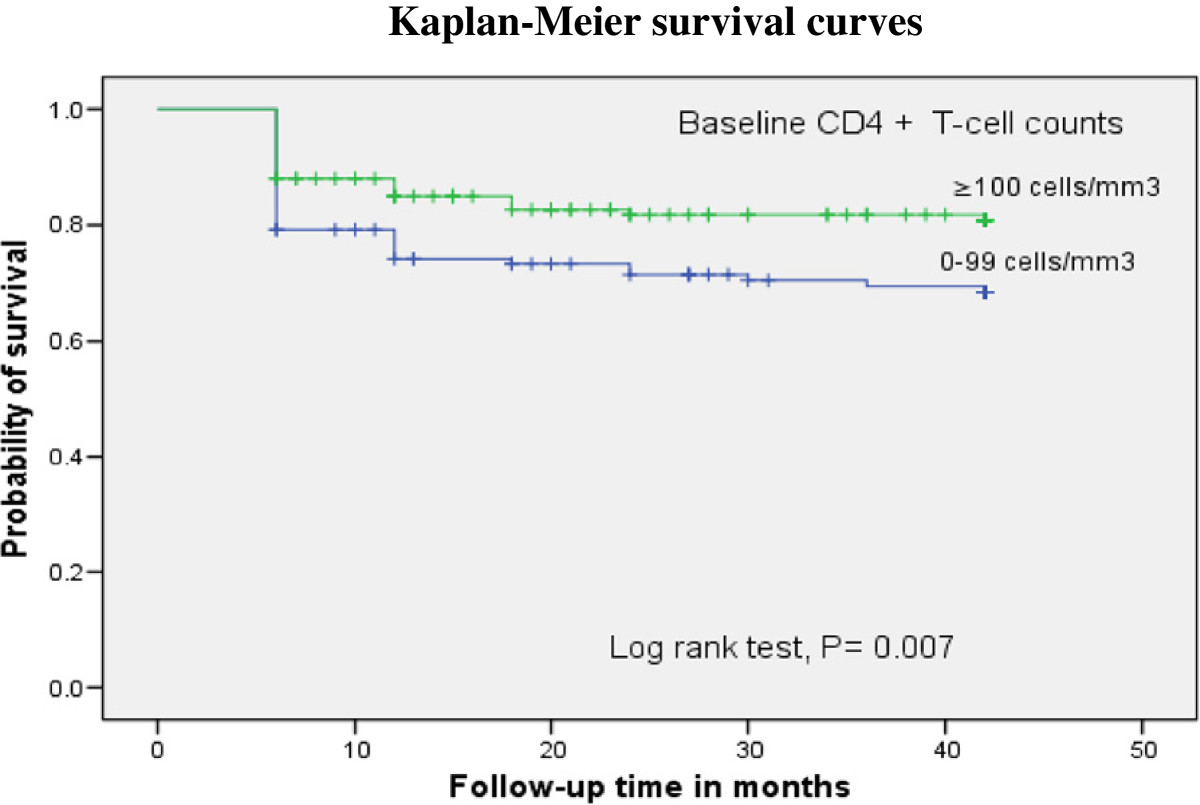
**Kaplan-Meier curve illustrating the probability of survival from immunological failure based on baseline CD4+ T-cell count.** The survival from immunological failure was lower in patients with baseline CD4+ T - cell count of 0–99 cells/mm^3^ as compared to those with CD4+ T - cell count of 100 cells/mm^3^ and above.

**Figure 4:**
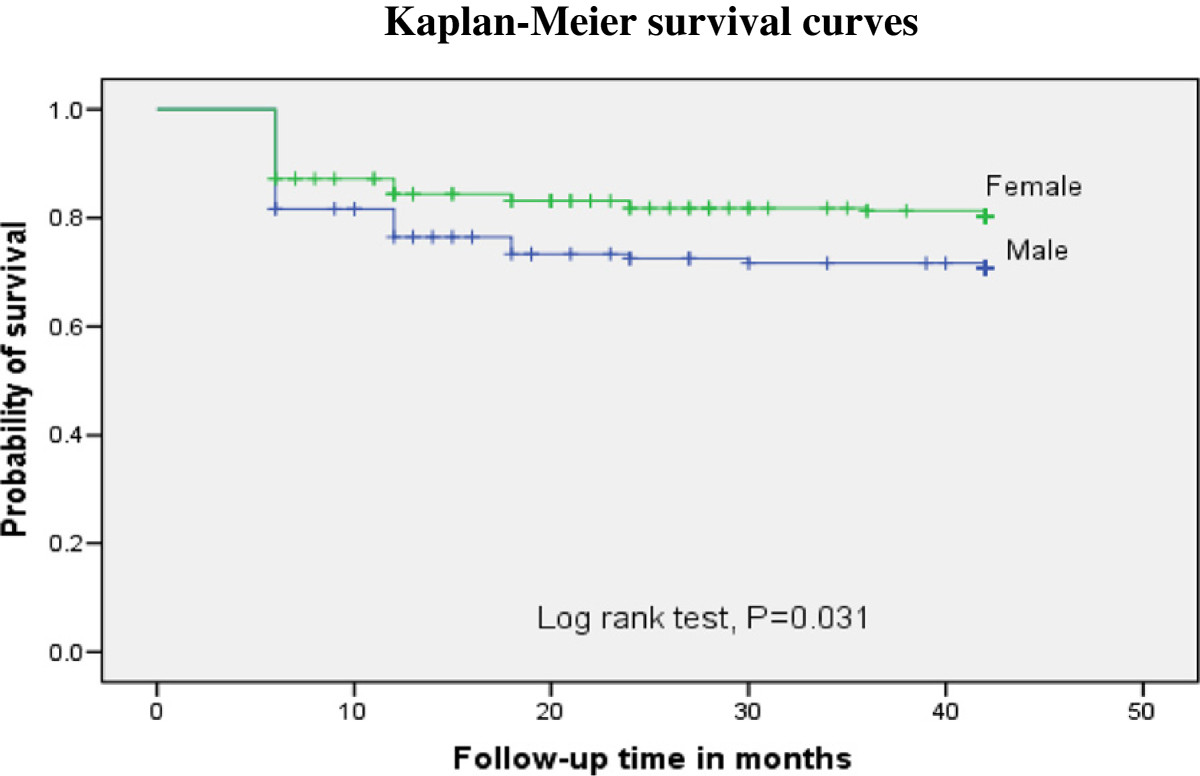
**Kaplan-Meier curve illustrating the probability of immunological failure survival to gender.** The survival from immunological failure was lower in male patients as compared to those female patients.

Kaplan Meier survival analysis showed that the probability of survival of patients from immunological failure was significantly lower among patients with baseline CD4+ T **-** cell count <100 cells/mm^3^ when compared to those CD4+ T **-** cell count of 100 cells/mm^3^ and above (log rank test, p = 0.007) (Figure 3). In addition, male patients showed lower immunological failure survival than females (log rank p = 0.031) (Figure 4).

In multivariate Cox regression analysis baseline CD4+ T **-** cell counts level <100 cells/mm^3^ (adjusted hazard ratio (AHR) 1.7, 95% CI: 1.11 **-** 2.64, p = 0.015) and being male sex (AHR 1.6, 95% CI: 1.01 **-** 2.37, p = 0.043) were found to be significant predictors of immunological failure. Even though the survival from immunological failure was significantly lower among patients with incident TB (p = 0.036), Cox regression analysis showed borderline significant association between incident TB and immunological failure (HR 1.9, 95% CI: 0.97 **-** 3.7, p = 0.06) (Table 3).

**Table 3:** **Cox-proportional hazard analysis of factors associated with immunological failure among HIV/AIDS patients who started ART from September 2007 to August 2008 at the University of Gondar Hospital**

Variables	Immunological failure		Crude HR (95% CI)	P	Adjusted HR (95% CI)	P
	No	Yes				
	no (%)	no (%)				
**Gender**						
Female	102(71.8)	40(28.2)	1		1	
Male	209(81)	49(19)	1.54(1.02-2.34)	0.042	1.6(1.01-2.68)	0.046
**Age (in year)**						
16-30	130(78.8)	35(21.2)	1			
31-50	164(77.7)	47(22.3)	1.06(.68-1.63)	0.812		
> 50	17(70.8)	7(29.2)	1.37(.61-3.09)	0.443		
**Education**						
Primary and below	175(76.8)	53(23.2)	1.12(.75-1.74)	0.546		
Secondary and above	136(79.1)	36(20.9)	1			
**WHO HIV clinical stage**						
I	47(82.5)	10(17.5)	1			
II	65(75.6)	21(24.4)	1.44(.69-3.06)	0.344		
III	161(77.4)	47(22.6)	1.31(.66-2.59)	0.438		
IV	38(77.6)	11(22.4)	1.32(.56-3.12)	0.525		
**Baseline CD4+ count (cells/mm** ^**3**^ **)**						
0-99	87(69.6)	38(30.4)	1.72(1.13-2.62)	0.012	1.8(1.10-2.92)	0.023
≥100	224(81.5)	51(18.5)	1		1	
**Initial ART regimen**						
D4T/3TC/NVP or EFV	40(81.6)	9(18.4)	1			
AZT/3TC/NVP or EFV	141(74.6)	48(25.4)	0.90(.43-1.89)	0.785		
TDF/3TC/NVP or EFV	130(80.2)	32(19.8)	1.29(.83-2.02)	0.262		
**Functional status**						
Working	262(77.1)	78(22.9)	1			
Ambulatory	39(79.6)	10(20.4)	1.44(.72-2.92)	0.301		
Bed ridden	10(90.9)	1(9.1)	1.26(.58-2.75)	0.555		
**Incident TB**						
Yes	16(61.5)	10(38.5)	1.92(.99-3.72)	0.050	2.2(0.94-5.09)	0.063
No	295(78.9)	79(21.1)	1		1	

d4T: stavudine; 3TC: lamivudine; NVP: nevirapine; AZT: zidovudine; EFV: efavirenz; TDF: tenofobir; TB: tuberculosis; HIV: human immunodeficiency virus; ART: antiretroviral therapy; IQR: inter-quartile range; SD: standard deviation; WHO: world health organization; HR: hazard ratio, P: p-value.

## Discussion

The baseline socio**-**demographic and immunological characteristics of this cohort were similar to other ART cohorts in sub-Saharan Africa, in which the majority of patients started ART at an advanced stage of the diseases and majority of the patients were females [6, 10, 12, 19]. In resource-limited settings evaluation of treatment outcomes mainly relies on immunological findings. Effective ARV therapy is usually convoyed by quantitative and functional restoration of patents’ immune response. Immunological failure may indicate incomplete suppression of plasma viral load [9]. In our study, 89 (22.2%) patients were experienced immunological failure within 42 months of follow-up. Of patients who have had immunological failure, 60(67.4%) patients were failed at 6 month of follow up. The immunological failure rate of our cohort was 8.5 per 100 patient-years. This finding is almost in line with the report from Debremarkos, Ethiopia, in which the immunological failure rate was 8 per 100 PY [19]. However, lower findings were reported from Latin America and Asia with failure rate of 2.57 per 100 PY and 1.1per 100 PY of follow **-** up respectively [20].

In our cohort, the immunological failure rate showed time-related downward trend with lowest failure rate occurred at the end of the follow-up. The majority of immunological failure in our cohort was occurred at 6 months of ART enrolment (30 per 100 PY) which is concordant with findings reported in other African countries [6, 19]. This might be due to the initiation of ART at an advanced stage of HIV/AIDS diseases. Baseline lower CD4+ T **-** cell counts associated with poor CD4+ T **-** cell count restoration and maintain long term lower immune response [10, 19, 21–23].

In this study, the log rank test showed that the immunological failure free survival proportion was significantly lower among patients with incident TB when compared to those patients remaining TB**-**free for the same follow**-**up time on ART. However, in the multivariate Cox regression analysis we observed borderline significant association between incident TB and immunological failure. This may be due to relatively small number of cases with incident TB. Different studies reported that despite TB treatment patients who developed TB after ART initiation were more likely to have lower immune recovery. Studies on South African [11] and Ugandan [12] cohorts of HIV patients initiated on ART, incident TB was associated with suboptimal CD4+ T **-** cell responses. Furthermore, a study from Senegal indicated that independent of HIV status development of incident TB causes severe CD4+ T**-**lymphocytopenia [17]. These findings may suggest that incident TB during ART is associated with long lasting immune suppression. Low baseline CD4+ T **-** cell counts at enrolment to an ART programme is associated with increased risks of TB and of mortality during the first year of ART [6, 7]. In this study the lower baseline CD4+ T **-** cell counts may expose patients to an increased risk of developing incident TB which can in turn contribute to immunological failure. Another explanation for the lower CD4+ T **-** cell recovery in patients with incident TB during ART may be due to decreased adherence to ART during TB treatment because of high pill burden and side-effects [24].

The most important finding of our study was that in multivariate Cox regression analysis lower baseline CD4+ T **-** cell counts were independently associated with immunological failure. This is supported by other studies conducted in Debremarkos, Ethiopia [19] and Thailand [21, 22] in which immunological failures were significantly associated with lower baseline CD4+ T **-** cell counts. Moreover, this finding is concordant with other reports where immune restoration is largely dependent on baseline CD4+ T **-** cell count and thus the timing of ART initiation is important in order to optimize the CD4+ T **-** cell response to therapy [23]. These reports may highlight that patients with low CD4+ T **-** cells count at baseline have poor long term CD4+ T **-** cells responses. Therefore, our finding supports the new WHO 2013 recommendations of expanding eligibility for ART initiation to CD4+ T **-** cell counts ≤500 cells/mm^3^ for all adults and children above 5 years [25].

Although the causal relationship is not known, keeping the report from Uganda [12] immunological failure was also associated with male sex. Advanced baseline WHO clinical stages and old age groups were significant predictors of poor immunological responses during ART [19]. However, in our study none of these factors were found to be associated with non-immunological responses of patients on ART. Unidentified factors such as opportunistic diseases and/or malnutrition might be the prime determinants of the immune recovery in the current study subjects.

### Study limitations

As a retrospective design our study had limitations. We excluded a large number of patient charts that could not be verified by chart review due to unavailability of full information which may have introduced bias. Moreover, because of the nature of the study design, it was difficult to control all possible confounders like body mass index and opportunistic infections other than TB. The sample size was also too small to include representative incident TB cases and with large sample size the effect of TB on immunological failure could be more elaborated. Given that virologic response is the preferred standard for monitoring ART, this study only assessed immunological responses of the patients and as a result that immunological failure could be related to viral resistance or non adherence. Therefore, for better understanding of the association between incident TB and CD4+ T **-** cell count recovery of HIV patients during ART prospective cohort study should be considered.

## Conclusion

High immunological failure rate in our cohort was occurred at six months of ART enrolment. The proportions of patients with impaired immune restoration were significantly higher among patients who developed incident TB during ART. Lower baseline CD4+ T **-** cell count of <100 cells/mm^3^ and being male sex were found to be significant predictors of immunological failure. The timing of initiation of ART was major determinants of the change in CD4+ T **-** cells count recovery. The result highlighted the beneficial effects of earlier initiation of ART on CD4+ T **-** cells recovery and strengthening early detection of TB through active screening and appropriate management of TB patients.

## Authors’ original submitted files for images

Below are the links to the authors’ original submitted files for images. Authors’ original file for figure 1 Authors’ original file for figure 2 Authors’ original file for figure 3 Authors’ original file for figure 4
