# Supplementary figures and images for: The effect of incident tuberculosis on immunological response of HIV patients on highly active anti-retroviral therapy at the university of Gondar hospital, northwest Ethiopia: a retrospective follow-up study
Source: BMC Infect Dis. 2014 Aug 27;14:468. doi: 10.1186/1471-2334-14-468 (PMC4158052; doi:10.1186/1471-2334-14-468)

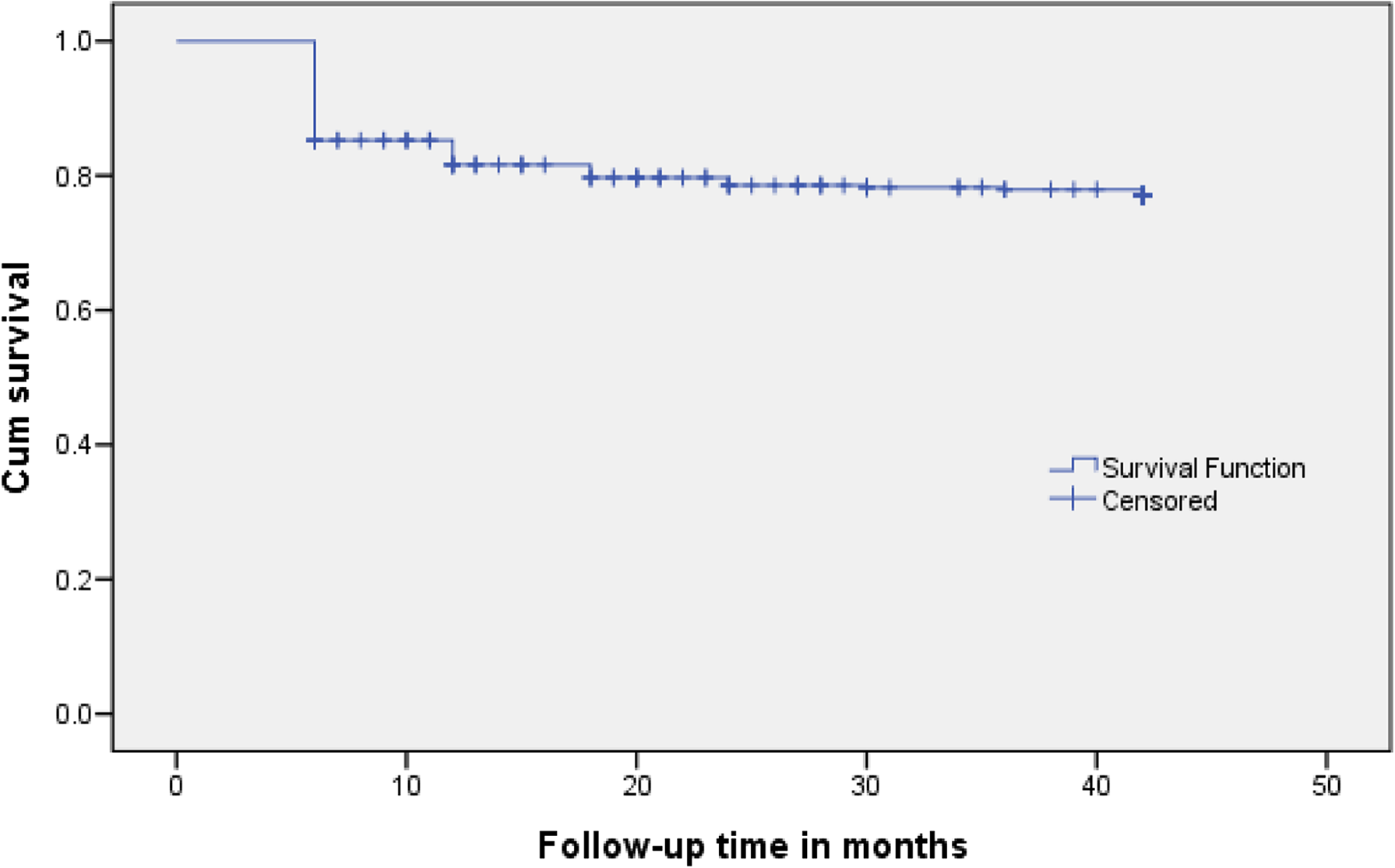

Supplement: Supplementary file 1 — Authors’ original file for figure 1 [file 12879_2014_3767_MOESM1_ESM.tif]

# Kaplan-Meier survival curves

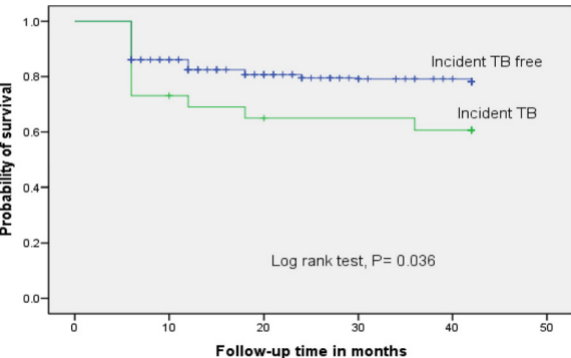

Supplement: Supplementary file 2 — Authors’ original file for figure 2 [file 12879_2014_3767_MOESM2_ESM.pdf]

# Kaplan-Meier survival curves

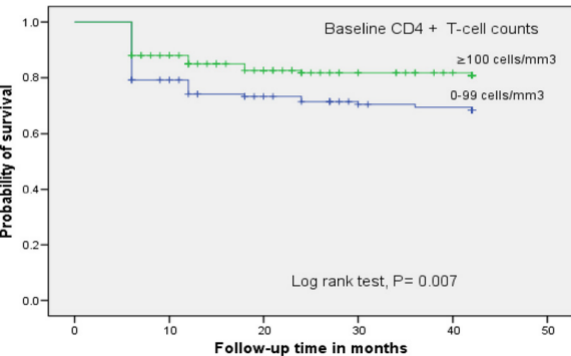

Supplement: Supplementary file 3 — Authors’ original file for figure 3 [file 12879_2014_3767_MOESM3_ESM.pdf]

# Kaplan-Meier survival curves

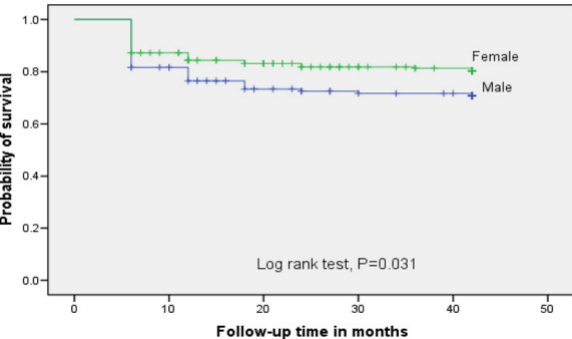

Supplement: Supplementary file 4 — Authors’ original file for figure 4 [file 12879_2014_3767_MOESM4_ESM.pdf]
